# Investigation of changes in protein stability and substrate affinity of 3CL-protease of SARS-CoV-2 caused by mutations

**DOI:** 10.1590/1678-4685-GMB-2021-0404

**Published:** 2022-04-29

**Authors:** Ekrem Akbulut

**Affiliations:** 1 Malatya Turgut Ozal University, Faculty of Engineering and Natural Sciences, Department of Bioengineering, Malatya, Turkey.

**Keywords:** 3CL-protease, mutation analysis, protein stability, SARS-CoV-2 genome, substrate affinity

## Abstract

3CL^pro^ of SARS-CoV-2 is one of the enzymes required for the replication process of the virus responsible for the COVID-19 pandemic. In this study, changes in protein stability and substrate affinity caused by mutations were investigated to stir the development of potent inhibitors. Sequence data of samples were obtained from the NCBI Virus database. Mutation analyses were performed with RDP4 and MegaX. 3CL^pro^ tertiary models were created using Robetta. Molecular docking for peptidomimetic substrate and inhibitor ligand was done with Autodock v4.2 and Haddock v2.4. Protein stability analysis was performed using mCSM stability and DynaMut2. Twenty-four missense mutations in 3CL^pro^ were identified in this study. Changes in the 3CL^pro^ structure induced by the mutations Met49Thr, Leu167Ser, and Val202Ala resulted in significant levels of instability (-2.029,-2.612,-2.177 kcal.mol^-1^, respectively). The lowest interaction energy for substrate was -58.7 kcal.mol^-1^ and -62.6 kcal.mol^-1^ in wild-type and mutant, respectively. The lowest docking energy for ligand was -6.19 and -9.52 kcal.mol^-1^ for wild-type and mutant, respectively. This study reports for the first time that mutations cause increased substrate affinity of 3CL^pro^ from SARS-CoV-2. This research provides important data for the development of potent peptidomimetic inhibitors for the treatment of COVID-19.

## Introduction

The etiologic agent of coronavirus disease 2019 (COVID-19), severe acute respiratory syndrome coronavirus 2 (SARS-CoV-2), has killed more than 4.97 million people worldwide (Wu et al., 2020; Worldometer, 2021). SARS-CoV-2 is the third zoonotic coronavirus outbreak after the emergence of SARS-CoV and the Middle East Respiratory Syndrome (MERS-CoV) in the last two decades. SARS-CoV-2 is a positive-sense RNA virus (Drosten et al., 2003; Zaki et al., 2012). The SARS-CoV-2 genome is noted for its high similarity to SARS-CoV and MERS-CoV (Akbulut, 2020a). Once the SARS-CoV-2 viral genome has entered the host cell, it is translated to yield two overlapping polyproteins (polyprotein1a and polyprotein1ab) (Zhu et al., 2020). 3-chymotrypsin-like protease (3CL^pro^) and papain-like protease (PL^pro^) contribute to the activation of 15 different non-structural proteins (nsp) by processing polyprotein1ab from 14 different points (Gao et al., 2021). The critical role of 3CL^pro^, a cysteine protease, in converting polyproteins into individual functional proteins for viral replication, as well as the enzyme’s highly conserved substrate selectivity among Coronaviruses, make it an attractive target for inhibitor screening (Su et al., 2020). The active site of 3CL^pro^ is sandwiched between two β-barrel domains, domain I (residue 10-99) and domain II (residue 100-182). Domain III (residue 198-306), forms a bundle of alpha-helices and is proposed to regulate dimerization (Douangamath et al., 2020).

Mutations can trigger changes in protein structure and stability, causing changes in protein functional properties, substrate/ligand affinity, and protein-protein interactions (Manolaridis et al., 2018; Kalathiya et al., 2019; Akbulut, 2021a, b).

Understanding the molecular-level mechanism of peptide cleavage catalyzed by cysteine proteases after mutations are crucial for designing strong structure-based inhibitors (Mengist et al., 2021). Here, changes in the structure and functional properties of 3CL^pro^ caused by mutations in the nsp5 of SARS CoV-2 were determined. To aid in the creation of efficient 3CL^pro^ inhibitors, structural alterations in proteins were studied.

## Materials and Methods

### Mutation data and protein stability analysis

Sequence data of 1,536,489 individuals were searched from the NCBI Virus database for the analysis of 3CL^pro^ mutations of SARS CoV-2. The study included 1,474 complete sequence data from the 2,950 sequence data from the Africa. Reference 3CL^pro^ accesion codes are YP_009725301_1 and NC_045512.2. Protein sequence information was aligned with the MAFFT (v7.487) multiple sequence alignment program FFT-NS-i algorithm (Katoh et al., 2018). The scoring matrix BLOSUM 80 and 1 PAM/k=2 was chosen for the amino acid sequences and nucleotide, respectively (Mount, 2008a,b). The gap opening penalty was used as 2.0. The mutated residues were analyzed with RDP4 and MegaX tools (Martin et al., 2015; Kumar et al., 2018). Analysis of changes in protein stability after the mutation was performed using mCSM stability and DynaMut2 (Pires et al., 2014; Rodrigues et al., 2021). The results were viewed with the NGL viewer (Rodrigues *et al.*, 2021).

### Mutant protein modeling

The homology model of the mutant 3CL^pro^ was created using Rosetta algorithms applying the deep residual neural network approach (Yang et al., 2020). 7K3T (PDB code) was selected as a template. ProSA was used for the structural quality of 3CL^pro^ models (Wiederstein and Sippl, 2007). Superimpose and conformational analyses of wild-type and mutant proteins were performed with PyMOL (ver2.4.1). Topological differences were calculated with the i-Tasser TM-Score and root mean square deviation (RMSD) algorithm (Xu and Zhang, 2010).

### Molecular docking

The wild-type and mutant of 3CL^pro^ were used as targets, and GC376 alpha-ketoamide analog-VR4 (CID 155804578) was used as an inhibitor ligand for molecular docking using AutoDock 4.2 (Steffen et al., 2010). Molecular docking was performed with a grid box of 70x74x80 (−11.333, −3.139, 5.833) with a grid spacing of 0.375 Å around the binding pocket. The simulations were performed with the Lamarckian genetic algorithm (LGA) (Morris et al., 1998). The LGA parameters were 100 runs, 2.7x10^4^ generations, and 500 population size. A maximum of 2.5x10^7^ energy evaluations was applied for each experiment. 

The change in substrate affinity after the mutation was tested with the protein-peptide docking using Haddock v2.4 (Van Zundert et al., 2016). The active site for the 3CL^pro^ was residues number 41,49,140, 144,145,163,165,166,168,172, and 189. Octa-peptide -SAVLQ/SGF- was used as a substrate to represent the nsp4/nsp5 cleavage site. The number of structures for rigid-body docking was set to 1000. The number of trials for rigid body minimization was set to 5. The number of structures for semi-flexible refinement was set to 200. Refined with short molecular dynamics in open solvent using water. The clustering method was selected Fraction of Common Contacts (FCC). RMSD cutoff for clustering was set to 0.6 Å. Kyte-Doolittle hydrophobicity scale method was used for solvating. Cutoff distance (proton-acceptor) to define a hydrogen bond was set to 2.5 Å. Cutoff distance (carbon-carbon) to define a hydrophobic contact was set to 3.9 Å. Docking parameters were performed as blind docking with default values. Docking results were visualized with Discovery SV (ver20.1, DDS Biovia).

## Results

**Figure 1-:**
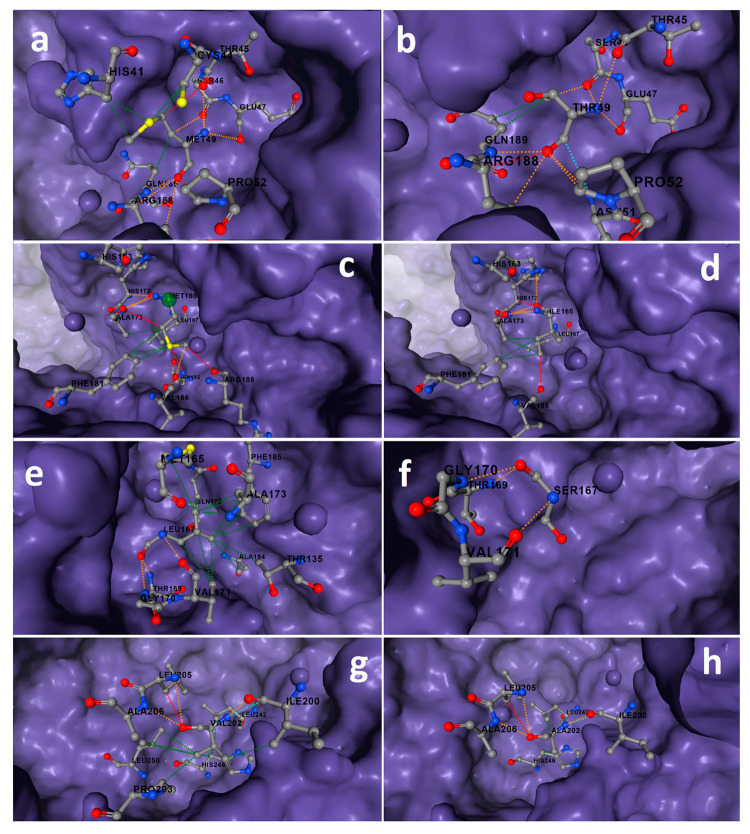
Surface-stick illustration of changes in protein stability induced by highly destabilizing mutations of 3CL^pro^ of SARS-CoV-2. a) Met49/wild-type, b) Thr49/mutant, c) Met165/wild-type, d) Ile165/mutant, e) Leu167/ wild-type, f) Ser167/mutant, g) Val202/wild-type, h) Ala202/mutant. Bond legends; red-hydrogen, green-hydrophobic, orange-polar, blue-van der walls, navy blue-carbonyl.

The results showed that a decrease in protein stability increased substrate and ligand affinity. Twenty-four missense mutations were detected in South African isolates of SARS CoV-2 in this study (Table 1). The protein tertiary structure of the mutant 3CL^pro^ was created with trRosetta, a deep neural network-based modeling algorithm. The Z-score of the homology model of mutant 3CL^pro^ was -7.36. The model was within the NMR quality limits. TM-score was 0.97. RMSD of the common residues was 1.601 Å. RMSD in superposition was 0.93 Å. Two of these mutations were shown to improve the stability of the 3CL^pro^ structure, whereas twenty-two were found to destabilize it. Changes in the 3CL^pro^ structure induced by the mutations Met49Thr, Leu167Ser, and Val202Ala resulted in significant levels of instability (-2.029, -2.612, and -2.177 kcal.mol^-1^, respectively) (Table 1). It was observed that the stable structure formed by Met^49^, which is involved in the stabilization of the active site with its strong bond network, in the wild protein with 5 hydrophobic, 7 polar interactions, and 2 hydrogen bonds, weakens in the mutant protein (Figure 1a,b). After the Met49Thr mutation, the hydrophobic interactions between the catalytic residue His^41^ and the Met^49^ residue were removed. After the Met165Ile and Leu167Ser mutations of Met^165^ and Leu^167^ residues, which play an important role in the formation of the substrate-binding pocket with their polar, hydrophobic, and Van der Walls interactions, significant weakening occurred in the interaction networks (Figure 1). The stable structure formed by Val^202^ in domain III, which is involved in dimerization, with five hydrophobic, three polar, one Van der Walls, and one hydrogen bond interactions were reduced to three polar, one Van der Walls, and one hydrogen bond interactions after mutation (Figure 1g,h). The Asn238Tyr/Ser mutations increased protein stability in its location. 

**Table 1 -:** Changes in stability of 3CL^pro^ of SARS-CoV2.

Mutant residue	Codon change	Charge change	DynaMut2	mCSM		
			ΔΔG (kcal.mol^-1^)	RSA (%)	ΔΔG (kcal.mol^-1^)	Output
Met49Thr	ACG > ATG	nP > uncP	-1.29	19.5	-2.029	Highly Destabilizing
Lys90Arg	AGG > AAG	nP > +	-0.25	56.5	-1.193	Destabilizing
Pro99Leu	CTT > CCT	nP > nP	-0.33	12.3	-0.58	Destabilizing
Met162Ile	ATT > ATG	nP > nP	-1.06	0.0	-1.299	Destabilizing
His164Leu	CTT > CAT	+ > nP	-0.75	0.0	-1.386	Destabilizing
Met165ILe	ATA > ATG	nP > nP	-1.34	8.4	-1.528	Destabilizing
Leu167Ser	TCA > TTA	nP > uncP	-2.23	0.4	-2.612	Highly Destabilizing
Pro168Ser	TCA > CCA	nP > uncP	-0.19	58.0	-0.464	Destabilizing
Thr169Ser	TCT > ACT	uncP > uncP	-0.01	85.0	-0.265	Destabilizing
Gly170Ala	GCA > GGA	nP > nP	-0.54	56.0	-0.568	Destabilizing
Gly195Ser	AGT > GGT	nP > uncP	-0.11	90.2	-0.354	Destabilizing
Gly195Val	GTT > GGT	nP > nP	-1.29	90.2	-0.351	Destabilizing
Thr196Ser	TCG > ACG	uncP > uncP	-0.32	61.1	-0.558	Destabilizing
Val202Phe	TTT > GTT	nP > nP	-1.69	13.2	-1.427	Destabilizing
Val202Ala	GCT > GTT	nP > nP	-1.97	13.2	-2.177	Highly Destabilizing
Asn203Tyr	TAT > AAT	uncP > uncP	-0.76	1.3	-1.274	Destabilizing
Tyr237Cys	TGC > TAC	uncP > nP	+0.27	29.2	-1.745	Destabilizing
Asn238Tyr	TAT > AAT	uncP > uncP	-0.32	72.1	+0.556	Stabilizing
Asn238Ser	AGT > AAT	uncP > uncP	+0.17	72.1	+0.273	Stabilizing
Tyr239His	CAT > TAT	uncP > +	-1.55	6.6	-1.871	Destabilizing
Tyr239Phe	TTT > TAT	uncP > nP	-0.99	6.6	-0.62	Destabilizing
Ala285Thr	ACT > GCT	nP > uncP	+0.17	73.2	-1.046	Destabilizing
Leu286Ile	ATA > TTA	nP > nP	-0.27	96.1	-0.488	Destabilizing
Phe305Val	GTC > TTC	nP > nP	-0.93	10.5	-1.41	Destabilizing

RSA-Residue relative solvent accessibility, nP-non polar, uncP-uncharged polar

**Figure 2-:**
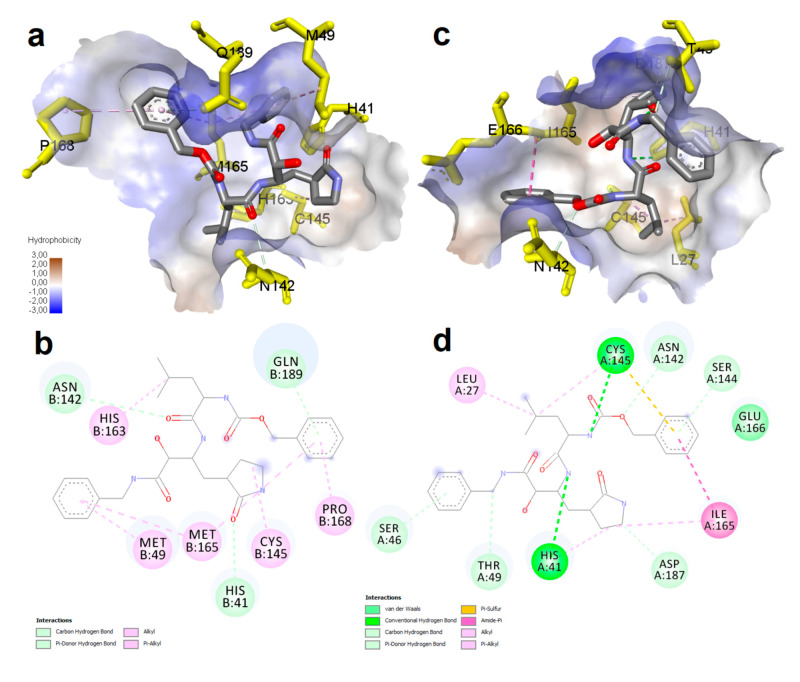
Representation of binding interaction of VR4 inhibitor ligand with 3CL^pro^ of SARS CoV-2. a) Hydrophobic surface illustration of wild-type 3CL^pro^-ligand complex, b) Diagram illustration of mutant 3CL^pro^-ligand complex, c) Hydrophobic surface illustration of wild-type 3CL^pro^-ligand complex, d) Diagram illustration of the mutant 3CL^pro^-ligand complex .

The results of mutations in molecular interaction were tested with molecular docking. The lowest binding energy for ligand was -6.19 kcal.mol^-1^ and -9.52 kcal.mol^-1^ for wild-type and mutant 3CL^pro^, respectively (Table 2). The minimum inhibitory concentration was 28.54 µM and 0.105 µM for wild-type and mutant 3CL^pro^, respectively. The wild-type 3CL^pro^ provided hydrogen bond interaction with the ligand at His41:CD2 - VR4:O14, Asn142:CA - A:VR4:O27, and Gln189:NE2 - VR4 positions, and hydrophobic interaction with residues Met^49^, Met^165^, Pro^168,^ and His^163^ (Figure 2). The mutant 3CL^pro^ provided hydrogen bond interaction with the ligand at VR4:N03 - Cys145:SG, VR4:N06 - His41:NE2, Asn142:CA - VR4:O32, VR4:C12 - Asp187:O, VR4:C18 - Thr49:OG1, Ser46:OG - VR4, and Ser144:OG - VR4 positions, and hydrophobic interaction with residues Leu^27^, His^41^, Cys^145^, Ile^165^,and Glu^166^. 

**Table 2-:** Docking results of wild and mutant 3CL^pro^ of SARS-CoV-2 with inhibitor ligand.

Row	wild				mutant			
	DocSc kcal.mol^-1^	Ki^e^ µM	Evdw	Eelec	DocSc kcal.mol^-1^	Ki^e^ µM	Evdw	Eelec
**1**	-6.19	28.54	-10.29	-0.08	-9.52	0.105	-13.68	-0.02
**2**	-5.78	58.17	-9.86	-0.09	-9.37	0.135	-13.57	0.02
**3**	-5.43	105.29	-9.51	-0.10	-8.90	0.298	-13.05	-0.02
**4**	-5.30	130.34	-9.47	-0.01	-8.67	0.440	-12.78	-0.07
**5**	-5.06	194.15	-9.13	-0.11	-8.61	0.488	-12.88	0.10
**6**	-5.05	197.66	-9.21	-0.02	-8.51	0.579	-12.65	-0.04
**7**	-5.01	212.89	-9.12	-0.06	-8.49	0.594	-12.85	0.18
**8**	-4.97	227.51	-9.09	-0.06	-8.39	0.703	-12.86	0.09
**9**	-4.92	247.09	-9.00	-0.10	-8.30	0.817	-12.44	-0.04
**10**	-4.88	263.91	-9.06	-0.03	-8.23	0.919	-12.5	0.09

DocSc-docking score, Ki^e^-estimated inhibitory constant, Evdw-van der walls energy, Eelec-electrostatic energy.

**Figure 3-:**
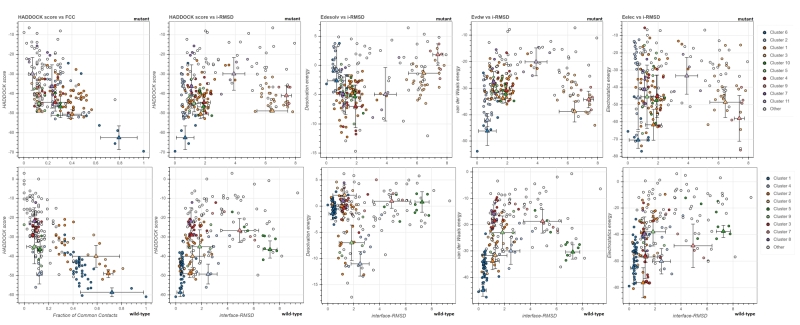
The quality parameters of 3CL^pro^-substrate interaction.

**Figure 4-:**
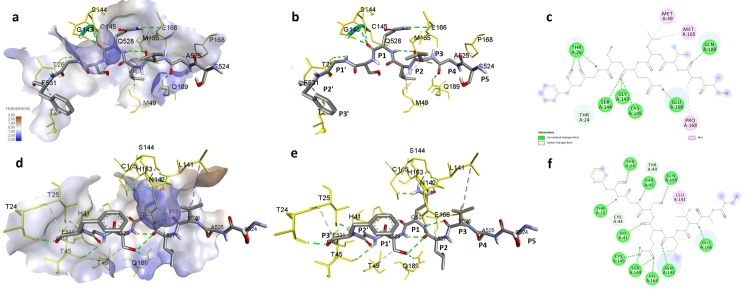
Representation of interaction of the substrate with 3CL^pro^ of SARS-CoV-2. a) Hydrophobic surface illustration of wild-type 3CL^pro^-substrate complex, b) Stick illustration of wild-type 3CL^pro^-substrate complex, c) Diagram illustration of wild-type 3CL^pro^-substrate complex, d) Hydrophobic surface illustration of mutant 3CL^pro^-substrate complex, e) Stick illustration of mutant 3CL^pro^-substrate complex, f) Diagram illustration of mutant 3CL^pro^-substrate complex.

Changes in protein structure induced by mutations resulted in increased interest in substrate affinity. FCC and interface-RMSD (i-RMSD) values indicate that the structure with the lowest energy differs significantly from other clusters and that Cluster-1 for wild-type and Cluster-6 for mutant were the correct binding model. While the lowest substrate interaction energy was -58.7 kcal.mol^-1^ in wild-type, the interaction was achieved with -62.6 kcal.mol^-1^ in mutant protein (Figure 3). Z-scores were -1.8 and -2.0 for wild-type/substrate and mutant/substrate complexes, respectively (Table 3). The interaction with the catalytic residues (His^41^ and Cys1^45^) was stronger in the mutant protein, and the formation of the substrate in the mutant protein binding groove was more successful (Figure 4).

**Table 3-:** Docking results of wild and mutant 3CL^pro^ of SARS-CoV-2 with the substrate.

		DocSc kcal.mol^-1^	i-RMSD	Evdw	Eelec	Edesolv	Z-Score
**1**	W	-58.7 +/- 2.0	0.5 +/- 0.4	-45.0 +/- 2.0	-65.0 +/- 10.1	-1.4 +/- 1.0	-1.8
	M	-62.6 +/- 5.2	0.3 +/- 0.2	-46.2 +/- 4.7	-70.6 +/- 4.2	-2.6 +/- 1.4	-2.0
**2**	W	-49.2 +/- 4.4	1.9 +/- 0.1	-29.9 +/- 4.4	-60.1 +/- 8.3	-11.0 +/- 2.0	-0.9
	M	-51.1 +/- 1.4	0.9 +/- 0.1	-42.0 +/- 2.1	-46.0 +/- 16.1	-0.7 +/- 3.4	-0.6
**3**	W	-49.0 +/- 1.8	0.5 +/- 0.0	-34.0 +/- 2.7	-76.3 +/- 9.8	-0.0 +/- 0.9	-0.9
	M	-50.5 +/- 1.9	1.0 +/- 0.2	-33.8 +/- 1.6	-61.7 +/- 7.8	-4.8 +/- 1.0	-0.6
**4**	W	-40.0 +/- 4.9	0.6 +/- 0.2	-31.7 +/- 4.3	-56.9 +/- 3.5	2.1 +/- 1.8	-0.1
	M	-48.9 +/- 0.5	2.1 +/- 0.1	-38.8 +/- 3.4	-48.6 +/- 7.7	-1.3 +/- 0.9	-0.4
**5**	W	-36.5 +/- 3.9	2.1 +/- 0.1	-30.4 +/- 2.4	-37.5 +/- 4.0	0.8 +/- 1.7	0.2
	M	-46.8 +/- 4.8	1.2 +/- 0.1	-32.2 +/- 2.8	-47.5 +/- 12.6	-5.4 +/- 1.7	-0.1
**6**	W	-35.1 +/- 7.6	1.6 +/- 0.3	-23.0 +/- 5.5	-37.9 +/- 9.3	-7.0 +/- 2.9	0.3
	M	-45.2 +/- 3.6	1.8 +/- 0.1	-31.1 +/- 2.1	-45.5 +/- 15.9	-5.0 +/- 3.0	0.1
**7**	W	-29.0 +/- 0.5	2.1 +/- 0.1	-18.5 +/- 1.8	-56.2 +/- 14.8	-0.4 +/- 1.5	0.8
	M	-44.5 +/- 3.9	1.2 +/- 0.1	-30.9 +/- 2.6	-45.2 +/- 10.6	-7.0 +/- 3.2	0.1
**8**	W	-26.7 +/- 5.3	2.1 +/- 0.1	-18.7 +/- 3.9	-48.4 +/- 14.2	1.0 +/- 1.2	1.0
	M	-41.1 +/- 4.4	2.1 +/- 0.0	-34.3 +/- 2.7	-58.0 +/- 11.5	1.8 +/- 1.6	0.5
**9**	W	-21.8 +/- 6.0	2.1 +/- 0.2	-15.9 +/- 3.0	-39.5 +/- 11.1	1.0 +/- 2.0	1.5
	M	-36.4 +/- 9.6	1.2 +/- 0.0	-27.6 +/- 3.4	-41.2 +/- 8.4	-4.9 +/- 2.5	1.1
**10**	W	-	-	-	-	-	-
	M	-29.9 +/- 7.2	1.7 +/- 0.2	-20.1 +/- 4.6	-33.3 +/- 9.2	-5.0 +/- 4.0	1.9

DocSc: docking score, i-RMSD: interface RMSD (from the overall lowest-energy structure), Evdw: Van der Waals energy, Eelec: electrostatic energy, Edesolv: desolvation energy, Eair: restraints violation energy.

## Discussion

SARS-CoV-2 will perhaps be one of the most devastating health problems of this century. Despite the success of vaccine studies to prevent the disease, the high mutation rate of SARS-CoV-2 made the current preventive efforts only partially successful, making the studies on the development of therapeutic drugs even more important. In this context, the most important drug targets are cysteine proteases, which provide the functional structure of the SARS-CoV-2 proteome. Cysteine proteases are attractive targets for covalent inhibitors (Kathman et al., 2014). In this study, changes in 3CL^pro^ stability and activity caused by mutations in South African isolates were investigated *in silico*. Mutations can cause changes in the structure and stability of drug target proteins, resulting in changes in substrate and ligand affinity (Akbulut, 2020b; Xavier et al., 2021). *In silico* studies have achieved great success in recent years due to their contributions to the design and development of effective drug molecules in a short time, taking these changes into account (Prabhavathi et al., 2020; Das et al., 2021). In this study, the data obtained showed that the decrease in protein stability due to mutations increased the affinity for the substrate and VR4 ligand, a cysteine protease inhibitor.

SARS-CoV-2 3CL^pro^ has two catalytic residues, His^41^ and Cys^145^. The residues 49, 140, 144, 163, 165, 166, 168, 172 and 189 are the main structures in the active site formation. The charge change near the active site can alter the energetic barrier to attaining oxyanion transition states (Simón and Goodman, 2010). The altered electrical charge and hydrophobic interactions as a result of mutations concentrated around the active site may explain the increased ligand and substrate affinity, and catalytic activity (Table 1). In dimerization, Glu^166^ gets close to the N-terminus of the protein. This movement contributes to the completion of the active site formation by providing the formation of the substrate specificity pocket and the oxyanion hole (Hilgenfeld, 2014). Conservation of the conformations of Glu^166^ and Phe^140^ involved in dimerization may indicate successful dimerization despite increased instability by mutations.

**Figure 5-:**
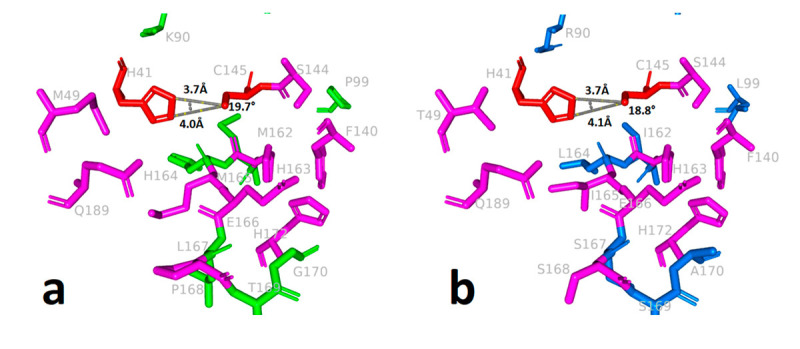
Conformational change in catalytic residues His^41^ and Cys^145^ a) wild-type 3CL^pro^, b) mutant 3CL^pro^.

The distance between His41:CE1 and Cys145:SG resulting in 0.1 Å divergence after mutation resulted in a narrowing of approximately 1 degree (0.9°) in the angle between His41:NE2-Cys145:SG and His41:CE1--Cys145:SG (Figure 5). The conformational structure and topology of the substrate-binding groove are one of the most fundamental factors determining the catalytic efficiency of 3CL^pro^. The changes in the substrate-binding groove and the changing topological structure caused by the mutations detected in South African isolates, most of which concern the active site residues and the surrounding area, may explain the increased activity. This might also explain the exponential increase in the number of instances after new mutations (Deparment Health Republic of South Africa, 2021).

The binding patterns presented in this study revealed a high degree of similarity with the high-resolution crystallographic substrate-binding patterns. (MacDonald et al., 2021). 3CL^pro^-substrate interactions contribute to fine-tuned substrate geometry that results in substrate-specific catalytic efficiency (Kneller et al., 2020). The point of interest here is whether SARS-CoV-2 functional proteins, organized by a mechanism that we can evaluate sequentially depending on the variability in substrate affinity, can act in coordination with increased protease activity. The differential affinity of 3CL^pro^ to nonstructural protein substrates for SARS-CoV-2 supports cleavage of pp1ab by a cascading mechanism. Despite the invariance of P1 (Gln) in substrates, the sequences before and after it are thought to play a role in programming this gradual cleavage by limiting the rate of catalytic activity (Snijder et al., 2016; Gildenhuys, 2020).

The process of formation of SARS-CoV-2 functional proteins begins with the autocleavage of 3CL^pro^. Phe305Val mutation detected in the C-terminal autocleavage site (Ser^301^-Gln^306^) increased unstable structure and motility in the autocleavage region (Table 1). Whether the Phe305Val mutation results in increased autocleavage activity is a further question to be answered.

The high instability caused by the Met49Thr mutation seen in the substrate-binding site results in increased substrate-enzyme interaction due to increased mobility. The S2 sub-binding site is formed by the Met^49^ and Met^165^ residues of 3CL^pro^. Mutations in Met49Thr and Met165Ile induced alterations in the active site’s conformation and topology (Figure 1a,b,c,d). 

Glu^166^ is one of the key residues in both dimerization and substrate binding (Świderek and Moliner, 2020; Ullrich et al., 2021). Unstable mutant neighboring residues (162,164,165,167, 168,169 and 170), which have a role in substrate localization, may be considered to be involved in the increase in substrate interaction of Glu^166^.

## Conclusion

This study was the first to report that mutations cause increased substrate affinity of 3CL^pro^ from SARS-CoV-2. Although mutations indicate increased substrate affinity and viral activity, the positional accuracy and increased affinity of the inhibitory ligand after mutations in the active site may also lead to increased success of therapeutic drugs. In addition, increased substrate affinity inspires efforts to use peptidomimetics without warheads as inhibitors against 3CL^pro^ of SARS-CoV-2.
